# Hypercalcemia Secondary to Antibiotic-Eluting Calcium Sulfate Beads

**DOI:** 10.7759/cureus.41661

**Published:** 2023-07-10

**Authors:** Rohan Ahuja, Samir Mehta, Sophia Galustian, Dorota Walewicz, Betty Drees

**Affiliations:** 1 Internal Medicine, University of Texas Health Science Center at Houston, Houston, USA; 2 Internal Medicine, University of Missouri Kansas City School of Medicine, Kansas City, USA; 3 Endocrinology, Crystal Run Healthcare, West Nyack, USA; 4 Endocrinology, Saint Luke's Hospital, Kansas City, USA; 5 Endocrinology, Diabetes and Metabolism, University of Missouri Kansas City School of Medicine, Kansas City, USA

**Keywords:** symptomatic, complication, antibiotic, calcium sulfate beads, hypercalcemia

## Abstract

The use of calcium sulfate beads (CSBs) as a carrier for local delivery of antibiotics is increasingly reported for the treatment of localized infections. They are used most commonly in bone and joint infections, post-trauma infections, diabetes-related foot wounds, and vascular grafts. Hypercalcemia is rarely reported with CSB use but is an important safety concern, and patients at higher risk should be identified prospectively and followed carefully postoperatively. This case report details an 85-year-old male who developed severe, symptomatic postoperative hypercalcemia after antibiotic bead placement in the right knee. He presented with confusion, weakness, and lethargy, and was subsequently treated with fluids, calcitonin, and alendronate. The patient quickly returned to normal mental status, and calcium levels normalized, leading to discharge. The case report and review of the literature describe an incident of severe hypercalcemia attributed to the use of antibiotic-eluting CSBs and describe the risk factors and time course that may be expected.

## Introduction

Calcium sulfate beads (CSBs) are biodegradable, delayed-release antibiotic carriers able to manage dead space and surgical site infections. The use of CSBs has increased significantly within the last few decades due to the 100% release of its antibiotic load, leading to increased sustained antibiotic concentrations [1,2]. However, complications have been noted, including transient hypercalcemia, wound drainage, and heterotropic ossificans [3]. Furthermore, prolonged symptomatic hypercalcemia has only been noted in a few cases [4,5]. This case adds to the existing literature and notes the importance of developing a better understanding of CSB implantation risk factors to prevent future incidents.

## Case presentation

An 85-year-old male was admitted to the hospital for right knee pain. His past medical history included coronary artery disease, pulmonary hypertension, obstructive sleep apnea, hyperlipidemia, stage 3 chronic kidney disease, osteoarthritis, gout, heart failure with preserved ejection fraction, benign prostatic hyperplasia, and atrial fibrillation. The patient’s medications included the following: amlodipine, allopurinol, acetaminophen, atorvastatin, aspirin, cholecalciferol 5,000 units one tablet by mouth daily, stool softener, gabapentin, hydrocodone, losartan, potassium chloride, tamsulosin, and trazodone. His surgical history included a total right knee arthroplasty 15 years before admission and total left knee arthroplasty six years before admission. He had the removal of the right knee prosthesis with the placement of an antibiotic spacer two months before admission. Evaluation of the right knee included cultures positive for multiple strains of staphylococci. He had pain after completing a course of oral antibiotics and was thus admitted for irrigation and debridement of the affected right knee.

The physical exam revealed a well-healed incision with no erythema in the right knee with a full range of motion and nontender calves. The patient was afebrile, had a pulse of 66 bpm, respiratory rate of 18, oxygen saturation of 98%, and elevated blood pressure of 175/60 mmHg. The knee procedure was performed on the day of admission with the placement of 20 ccs of Stimulan antibiotic-eluting CSBs made of vancomycin and tobramycin in the right knee without complication. Preoperative laboratory results demonstrated a creatinine of 1.7 mg/dL, an estimated glomerular filtration rate of 38 ml/min/1.73sqm, white blood cell count of 10.83 TH/ul, hemoglobin of 11.3 g/dL, hematocrit of 34%, and a platelet count of 414 TH/uL. Immediate preoperative calcium and renal function were not obtained, as the initial postoperative laboratory assessment was normal. Of note, prior to surgery, calcium levels were not obtained but were normal two months prior ranging from 8.6 to 9.8 mg/dL. The postoperative laboratory results are shown in Table 1.

**Table 1:** Postoperative laboratory evaluations

Postoperative day	Corrected calcium, mg/dl (normal: 8.5-10.4 mg/dl)	Albumin, g/L (normal: 3.4-5.4 g/L)	Phosphorus, mg/dl (normal: 2.8-4.5 mg/dl)	25-OH vitamin D, ng/dl (normal: 20-40 ng/dl)	1,25-(OH)^2^ vitamin D, pg/ml (normal: 25-40 pg/ml)	Parathyroid hormone, pg/ml (normal: 10-55 pg/ml)
1	9.9					
2	11.8					
3	13.5					
4	13.7		4.6			
5	14.4	1.9	3.1			
6	11.9	2.4	2.9	51	21.9	15
7	10.5	2.2	2			
8	10.5	2.4	3.2			
9	10.9	2.6	2.2			
10	10.0	2.2	3.5			
11	9.9	2.2	3.4			
12	9.6	2.6	3.6			
13	9.4	2.7	2.4			
14	9.4	2.8	2.8			
15	8.9	2.7	3.1			

**Figure 1:**
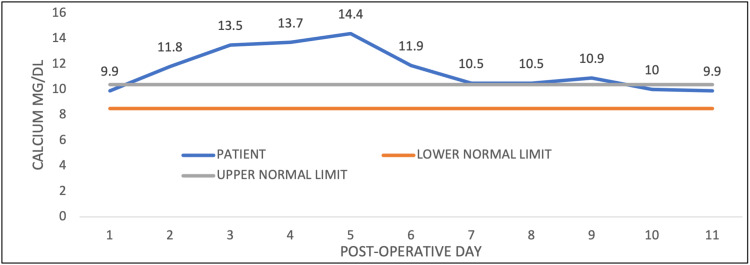
Calcium levels on each postoperative day Postoperative calcium levels compared to normal reference ranges. The hypercalcemia peaked on day five and returned to normal by day 10.

The patient began to become progressively more confused, and by postoperative day four, he became lethargic, could not follow commands, and was non-communicative. At that time, his serum calcium (corrected for hypoalbuminemia) was 14.4 mg/dL. Additional laboratory evaluations, including parathyroid hormone, vitamin D, albumin, and phosphorous levels, on postoperative day six, are reported in Table 1. Serum protein electrophoresis was negative for evidence of underlying multiple myeloma. The hypercalcemia of 14.4 mg/dL was treated with saline hydration, 24 hours of subcutaneous calcitonin, and one dose of intravenous pamidronate on postoperative day five. His calcium levels declined, and his mental status gradually improved. Changes in the patient's serum calcium are demonstrated by the graph in Figure 1. He was discharged on postoperative day 18 with a serum calcium level of 8.2 mg/dL and baseline creatinine of 1.8 mg/dL. No additional cause of acute hypercalcemia was identified during the hospitalization.

## Discussion

This case represents a rare but serious complication of severe hypercalcemia associated with antibiotic-eluting CSBs. This occurrence has been shown in a few case reports and safety reviews of CSB in the setting of joint infection. When hypercalcemia occurs in the setting of antibiotic-eluting CCBs, it generally starts on the second postoperative day, peaks at approximately day five, is rarely symptomatic, and resolves spontaneously. However, severe, symptomatic, and prolonged hypercalcemia may occur [1-5]. The patient’s complex history including chronic polypharmacy along with dehydration likely contributed to impaired renal calcium excretion. Both factors with the implantation of CCBs significantly increased his risk for hypercalcemia, although the volume of CCBs (20 ccs) in this case was lower than the volumes reported in the literature associated with higher risks (40 ccs) [3]. Although no preoperative acid-base disorders were present, the patient’s multi-faceted history predisposed him to symptomatic hypercalcemia. He responded well with supportive calcitonin and bisphosphonates, returning to normal serum calcium in 10 days.

CSBs are a synthetic hemihydrate form of calcium sulfate, biocompatible hydrophilic crystals that become soft after hydration and disappear two days after implantation into a joint. They are primarily used for peri-prosthetic joint infection as a synthetic carrier for antibiotic treatment [6]. A volume of 10 ccs of CCB contains 20 g of CaSO4, which is 5.73 g of elemental calcium, which is absorbed over a period of three to 12 weeks [7]. Studies of the antibiotic elution profile show antibiotic peak at 24-48 hours, but sustained levels of antibiotics over several weeks [8]. The elution rate of antibiotics and absorption rate of the calcium sulfate may depend on the implantation site, vascularity, the volume of beads, size of beads, the other agents mixed with the beads, etc. An extensive systemic review of CCBs was published by Abosala et al. in 2020. Complications are rare but include hypercalcemia (5.4%), heterotrophic ossificans (1.7%), and increased wound discharge [1]. Aside from the volume of bead implanted, individual patient factors contribute to the risk of hypercalcemia. A prospective observational study on the safety of CCBs was reported in 2018 by Kallala et al. on a series of 755 patients undergoing lower limb revisions. Of the 41 (5.4%) patients who developed hypercalcemia, only two were symptomatic. They noted increased risk with an increased volume of CCBs implanted and recommend a maximum of 40 cc [3]. The patient being symptomatic represents, especially at a lower dose, a point of distinction in the current literature as out of thousands of bead implantations, only three cases of symptomatic hypercalcemia are present. Additionally, in one of those cases, 40 ccs of CSB was given, redemonstrating the unique nature of the patient's presentation.

## Conclusions

Antibiotic-eluting CCBs are an effective therapy for many patients with bone and other infections. Although severe, symptomatic hypercalcemia is a rare complication of antibiotic-eluting CCBs, and the increasing use of CCBs is predicted to increase the number of cases. Physicians caring for these patients pre- and postoperatively should be aware of this potential complication, especially in patients who may have impaired renal capacity to clear the calcium load due to pre-existing chronic kidney disease, metabolic alkalosis, age, interfering medications (e.g. thiazide diuretics), or other toxins (e.g. myeloma proteins). Calcium levels, renal function, and hydration status should be monitored closely and volumes of CCBs limited in patients at higher risk.
